# Factors associated with pregnancy and induced abortion among street-involved female adolescents in two Nigerian urban cities: a mixed-method study

**DOI:** 10.1186/s12913-022-09014-x

**Published:** 2023-01-11

**Authors:** Mary O. Obiyan, Atinuke O. Olaleye, Funmilola F. Oyinlola, Morenike O. Folayan

**Affiliations:** 1 Department of Demography and Social Statistics, Obafemi Awolowo University, Ile-Ife, Nigeria; 2 Department of Obstetrics and Gynecology, Babcock University, Ilishan, Nigeria; 3 Department of Child Dental Health, Obafemi Awolowo University, Ile-Ife, Nigeria

**Keywords:** Teen pregnancy, Female adolescents, Induced abortion, Street-involved young people, Nigeria

## Abstract

**Objectives:**

This study determined the correlates of unwanted pregnancy and induced abortion among sexually active female street-involved adolescents (SIAs) aged 10–19 years in two urban cities in South-west, Nigeria.

**Methods:**

The data for this study were extracted from a larger mixed-method survey dataset on the sexual and reproductive health (SRH) of 1505 street-involved young people aged 10 to 24 years. For the quantitative data, the explanatory variables were age, history of school attendance, employment status, religion, living arrangement and city of residence. The study outcomes were a history of pregnancy and a history of induced abortion of last pregnancy. Binomial regression analysis was performed to determine the association between the explanatory and outcome variables. For the qualitative data generated through focus group discussions and in-depth-interviews, inductive and deductive approaches were used in conducting a thematic analysis to explore the perspectives and experiences of SIA on pregnancy and induced abortion.

**Results:**

Of the 424 female SIAs, 270 (63.7%) reported having had sex. Sixty-four (23.7%) respondents had a history of pregnancy, of which 38 (59.4%) gave a history of induced abortion of the last pregnancy. A history of school attendance significantly reduced the likelihood of being pregnant (AOR: 0.42, 95% C.I: 0.19–0.91), while 15–19-years-old SIAs who were pregnant were significantly less likely to abort (AOR: 0.13, 95% C.I: 0.02–0.77). Qualitative reports indicated that unintended pregnancy and induced abortion was a common experience among the sexually active SIAs. Many participants were aware of the methods of, and places to induce abortion.

**Conclusion:**

A large proportion of SIAs are sexually active with a high incidence of unintended pregnancy and a high rate of unsafe abortion. Access of female SIAs to education can reduce the risk of unintended pregnancy. Attention needs to be paid to how SIAs can have access to contraception.

## Background

Unsafe abortion, defined as a *procedure for terminating a pregnancy performed by persons lacking the necessary skills or in an environment that does not conform to the minimal medical standards, or both* [1]*,* mostly result from unintended pregnancies. From 2010 to 2014, an estimated 25 million unsafe abortions occurred annually worldwide [2], and 8 million were carried out in unsafe conditions [3], and most were in developing countries. Also, 15% or 3.2 million of the 21.2 million unsafe abortions were among adolescents aged 15–19 years [4, 5]. The prevalence of unsafe abortion is high in countries with restrictive abortion laws [6], many of which are in low- and middle-income countries that also limit the access of adolescents to contraception [5]. These restrictive laws have negative consequences – about half of the pregnancies among female adolescents aged 15–19 was unintended, and a higher proportion of these pregnancies end in unsafe abortions [7]. Africa has the highest number of maternal deaths due to unsafe abortion [8] despite the availability of procedures to prevent nearly all abortion-related deaths and disabilities [9].

The United Nations Sustainable Development Goal 3.7 explicitly requires that all States ensure their citizens have universal access to sexual and reproductive healthcare services integrated into the national strategies and programs by 2030 [10]. Some of these sexual and reproductive health services include contraception, sexual and reproductive health information and education [11]. Women’s sexual and reproductive health is linked to the achievement of goals 1, 2, 4, 5, 8, 9, 10, 11 and 17 of the sustainable development goals [12]. Thus, the continued neglect and inequality in access of female adolescents to sexual and reproductive health (SRH) services, especially in resource-constrained settings, is a threat to sustainable development goals.

A fundamental SRH need for adolescents is the prevention of unwanted pregnancies [13]. Barriers to accessing these services include the absence of SRH services for adolescents, and where there are services there are multiple structural and individual barriers to accessing these services [14, 15]. Also, where adolescents are managed as a homogenous group, the likelihood that interventions may fail is high [16]. Recognising the diversity in the population of adolescents helps to ensure interventions and service delivery strategies are tailored to the needs of different sub-populations of adolescents [17].

A sub-population of adolescents are street-involved adolescents (SIAs). SIA make their living and/or their homes on the streets [18]. Street-involved adolescents are boys and girls for whom the street in the widest sense of the word … has become their habitual abode and/or source of livelihood, and who is inadequately protected, supervised, or directed by responsible adults [19] SIAs are often left behind in the planning of SRH interventions [20]. SIAs tend to have very early sexual debut [21, 22] and are at high risk for sexual abuse and sexual exploitation [23]. There is no information on the population of street-informed adolescents in Nigeria. However, the majority are sexually active, have multiple sex partners and condom use is low [24]. Though the knowledge about modern contraceptives reduced the risk for inconsistent condom use among street kids in Nigeria, just about 3 in 10 sexually active street kids know about modern contraceptives [25]. Counselling and SRH information and services are grossly inadequate or unavailable for many SIAs, while social stigmatization and marginalization make it challenging to access the services where they exist [26, 27].

Various biological, cultural, psychosocial and structural factors increase the vulnerability of female SIAs to SRH problems [28]. The paucity of evidence on the SRH needs of SIAs poses a challenge for developing and evaluating programs that would promote their access to contraception services [29] in Nigeria. We designed a population-level study to determine the factors associated with pregnancy for female street-involved adolescents (SIAs) aged 10–19 years in two urban cities in South-west, Nigeria. We also determined factors associated with the induced abortion of the last pregnancy by those with a history of pregnancy.

## Methods

### Ethical considerations

The study’s ethics approval was obtained from the Institute of Public Health Research Ethics Committee, Obafemi Awolowo University Ile-Ife (IPHOAU/12/1133). Additionally, ethics approval was obtained from Osun State (OSHREC/PRS/569 T/154) Health Research Ethics Committee and social approval (LSMH2695/11/260/T) from the Lagos State Ministry of Health. The study was conducted in line with the declaration of Helsinki. Informed written consent was obtained from all study participants. Informed parental consent was waived for children below 18 years from both ethics’ committees in line with the national guidelines on sexual and reproductive health research conduct with adolescents [30]. This was because the study was non-invasive, and the children were street kids with associated challenges of locating parents for study approval purposes.

### Study design

This study is a secondary data analysis of a primary concurrent mixed-method baseline survey on SRH of young people aged 10 to 24 years who spent most of their time on the street (street-involved young persons). The survey was conducted between January and February 2019 in two urban cities – Lagos and Osogbo - located in south-west Nigeria. The study sites were purposefully selected: SIA are less likely to be found in rural communities, so a rural community was not considered for SIA recruitment. SIA were recruited from two urban areas with likely different profiles: Lagos, which is a cosmopolitan city, and Osogbo which is an urban settlement. The clusters of SIA in Lagos State were recruited from Bariga and Ajah, while that for Osun State were Oke-Baale and Olaiya/Sabo which are officially recognised sites where there are large clusters of SIA.

This study employed respondent-driven sampling and time-location sampling to recruit respondents. Quantitative and qualitative data were collected concurrently from study participants to provide comprehensive and richer information on SRH knowledge, practices and the associated factors. The quantitative data provided information on the prevalence of unintended pregnancy, induced abortion and their associated factors among respondents, while the qualitative data explored the perspectives of SIAs on pregnancy and induced abortion and their lived experiences. Details of the methodology of the primary study have been reported [25].

### Study population and sample

#### Sample size

The primary study recruited 1505 male and female 10–24 years old street-involved young people who either live “on the street” (working on the streets and return home to families at night) or live “of the street” (they never return home or have no contact with any family member) [18, 31]. The data for a total of 424 female adolescents aged 10–19 years were extracted for this study.

#### Explanatory and outcome variables

The explanatory variables for this study were the socio-demographic characteristics (age in grouped years, history of school attendance, working for pay, the importance of religion, living arrangement and city of residence). The outcome variables were a history of pregnancy and history of induced abortion of the last pregnancy. SRH knowledge (access to information on SRH, knowledge of modern contraceptives) and use of any modern contraceptives were controlled variables in the analysis.

Age was defined as age at last birthday and grouped into 10–14 (young adolescents) and 15–19 (older adolescents). Respondents were required to answer the following questions: ever attended school (yes/no), current working status – if working for pay – (yes/no), level of importance of religion as a personal value (very important/less important), living arrangement (alone, living with both parents, living with mother alone, living with father alone), and city of residence in the last 5 years (Lagos/Osun).

Access to information on SRH was determined from the responses to the question, ‘ever attended/or were given a talk on SRH’; a positive response was assigned “1”, and otherwise “0”. Knowledge of modern contraceptives was deduced from respondent responses to the question - “do you know of any of these methods which men and women can use to prevent pregnancy?” There were seven response options: (i) injection, (ii) condom, (iii) emergency contraception, (iv) traditional method, (v) withdrawal method, (vi) safe period and (vii) periodic abstinence. Respondents who picked options i = iii were categorized as having knowledge of modern method of contraception (1). Those who picked only options iv-vii were categorized as not having knowledge of modern contraception (0).

The respondents were asked to state the contraceptive method they used at last sexual activity with the current sexual partner. The response options included a list of modern and traditional contraceptive methods, an option that states ‘do not remember’ and an option that stated ‘I have not had sex’. All respondents with options other than ‘I have not had sex’ were categorized as sexually active.

Respondents who were sexually active were also asked if they had ever been pregnant. The response options were ‘yes’ if they had ever been pregnant, or ‘no’ if they had never been pregnant. Pregnancy was determined as having missed one’s menstrual period and having a confirmed positive pregnancy test. Those who gave a positive pregnancy response were further asked a question on the outcome of the last pregnancy. The response options were ‘currently pregnant’, ‘induced abortion’, ‘spontaneous abortion’, and, ‘livebirth’. Only respondents who reported the outcome of the last pregnancy as induced abortion were included in the analysis.

#### Data collection and analysis

An interviewer-administered tool was used to collect data from study respondents. The tool was pre-tested among purposively sampled study populations in locations different from the study areas, and corrections were made to worded and ambiguous questions before the main survey exercise. The tool was translated to the local dialect of the study areas – Yoruba. There were field workers that spoke other Nigerian languages who could interpret key concepts using the other two major languages - Igbo and Hausa.

The survey tool had twelve sections namely: socio-economic and demographic factors, sources of information and knowledge about reproductive health, sexual health practices, and knowledge and use of modern contraceptives. Others were knowledge and access to SRH services, homosexual experience, knowledge of HIV/AIDS and other sexually transmitted diseases, knowledge and attitudes to condom use, sexuality, gender and gender norms, use and perception of health services, and quality of life.

Data were collected by trained field workers using an electronic app – REDCap (v4.2.5- Vanderbilt University), which is a secure data collection web application used to build and manage online surveys/databases. The analysis was conducted using Stata SE 15.1 (Stata Corporation, College Station, Texas). The percentage distribution of participants by explanatory variables was determined. The test of associations between the explanatory and outcome variables was also conducted using the Pearson chi-square test. Binomial regression analysis was performed to determine the risk indicators for a history of unwanted pregnancy and a history of induced abortion. Adjusted and unadjusted models were developed for the analysis. These study variables fulfilled the underlying assumption of binomial regression, and the model fit carried out using the Hosmer-Lemeshow test was positive for the two models.

### Qualitative study

#### Study procedure

The qualitative study explored the perspectives and SRH experiences of street-involved young people. Participants who completed the interviewer-administered questionnaire were approached to participate in a focus group discussion (FGD) or an in-depth interview (IDI) a few hours later. A comfortable and private place was used for the FGD and IDIs to ease communications during discussions.

The FGD was stratified by age (10–14 years, 15–19 years, 20–24 years) and gender (male, female). Three FGDs were conducted for female SIAs: one for 10–14-year-olds and two for 15–19 years old. Each FGD had about 10–11 participants in each group, sessions lasted between 30 and 52 minutes, and all sessions were conducted in the local dialect. A trained interviewer moderated each FGD and a note-taker was present. Discussions and interviews were audio-recorded, and handwritten notes were taken. A total of 33 SIAs (FGD = 29, and IDI = 4) participated in the qualitative phase of the study.

#### Data analysis

All transcripts were translated from the local dialects to the English language by professionals, while the translations were further reviewed by another professional to ensure that contextual words, expressions and meanings are retained. The English transcripts were categorized using inductive line by line coding which was then built into themes. Line-by-line coding is a stage of open-coding that allows phenomena or concepts to be inductively recognized [32, 33]. Inductive coding allows themes to emerge from the data and allows meanings of the phenomenon to be uncovered [34, 35]. After arriving at some important themes, we gradually moved to deductive coding to address study aims. The team of eight persons developed the codes, themes and sub-themes. Both inductive and deductive thematic analysis was conducted using NVivo 12.

## Results

### Quantitative results

Table 1 highlights the sociodemographic profile of the study participants. The mean age of respondents was 15.59 and most participant had a history of school attendance (72.9%). A higher proportion were unemployed (75.0%), claimed religion was very important to them (62.0%), lived with both parents (50.9%) and reside in Osun State (51.4%).

**Table 1:** Socio-demographics of female street-involved adolescents (*N* = 424)

Variables	Frequency n (%)
**Age Group**	**Mean Age: 15.59**
10–14	137 (32.3%)
15–19	287 (67.7%)
**Ever attended school**	
No	115 (27.1%)
Yes	309 (72.9%)
**Work for Pay**	
No	318 (75.0%)
Yes	106 (25.0%)
**Importance of religion**	
Very important	263 (62.0%)
Less important	161 (38.0%)
**Living Arrangement**	
Alone	141 (33.3%)
Live with father alone	25 (5.9%)
Live with mother alone	42 (9.9%))
Live with both	216 (50.9%)
**Residence**	
Lagos	206 (48.6%)
Osun	218 (51.4%)

Table 2 highlights the proportion of study participants who had access to SRH information and their history of pregnancy and abortion. Eight of every ten SIAs had no access to SRH information (84.2%), and most (74.5%) were not aware of a modern contraceptive method. Six of every ten (63.7%) were sexually active at the time of the survey, and four of every five (83.0%) sexually active SIAs did not use a modern method of contraception at the last sexual intercourse. Of the 270 sexually active respondents, 64 (23.7%) had a history of pregnancy, of which 38 (59.4%) had induced abortion of their last pregnancy, 17 (26.6%) were currently pregnant, 2 (3.1%) had a spontaneous abortion of their last pregnancy, and 7 (10.9%) had a live birth.

**Table 2:** Access to SRH information, use of modern contraception, and history of pregnancy and induced abortion among female street-involved adolescents in Nigeria (*N* = 424)

Variables	Frequency
**Access to SRH Information**	
No	357 (84.2%)
Yes	67 (15.8%)
**Know modern contraceptives**	
No	316 (74.5%)
Yes	108 (25.5%)
**Sexually active**	
No	154 (36.3%)
Yes	270 (63.7%)
**Use of modern contraceptives at last sexual intercourse (*n*** **= 270)**	
No	224 (83.0)
Yes	46 (17.0%)
**History of pregnancy (*n*** **= 270)**	
No	206 (76.3%)
Yes	64 (23.7%)
**Outcome of last pregnancy (*n*** **= 64)**	
Currently pregnant	17 (26.6%)
Induced abortion	38 (59.4%)
Spontaneous abortion	2 (3.1%)
Live birth	7 (10.9%)

Table 3 shows the variables associated with the history of pregnancy and induction of abortion for the last pregnancy. Among the 64 respondents that reported ever being pregnant, a higher proportion were aged 15–19 years old (24.5%), did not attend school (29%), were employed (28.2%), lived with both parents (26.5%) and were resident in Osun State (25.3%).

**Table 3:** Association between explanatory variables, history of pregnancy and induced abortion of last pregnancy among female SIAs in Nigeria

Variables	History of Pregnancy			Induced Abortion of last pregnancy		
**Age group**	Yes (*n* = 64)	No (*n* = 206)	*p*- value	Yes (*n* = 38)	No (*n* = 26)	*p*- value
10–14	17 (21.8)	61 (78.2)	*P* = 0.64	14 (82.4)	3 (17.7)	*P* = 0.02*
15–19	47 (24.5)	145 (75.5)		24 (51.1)	23 (48.9)	
**Ever attended school**						
No	20 (29.0)	49 (71.0)	*P* = 0.23	13 (65.0)	7 (35.0)	*P* = 0.54
Yes	44 (21.9)	157 (78.1)		25 (56.8)	19 (43.2)	
**Work for pay**						
No	44 (22.1)	155 (77.9)	*P* = 0.30	28 (63.6)	16 (36.4)	*P* = 0.30
Yes	20 (28.2)	51 (71.8)		10 (50.0)	10 (50.0)	
**Importance of religion**						
Very important	39 (23.1)	130 (76.9)	*P* = 0.75	24 (61.5)	15 (38.5)	*P* = 0.66
Less important	25 (24.8)	25 (24.8)		14 (56.0)	11 (44.0)	
**Living Arrangement**						
Alone	21 (22.6)	72 (77.4)	*P* = 0.61	13 (61.9)	8 (38.1)	*P* = 0.95
Live with father alone	4 (22.2)	14 (77.8)		2 (50.0)	2 (50.0)	
Live with mother alone	4 (14.8)	23 (85.2)		2 (50.0)	2 (50.0)	
Live with both	35 (26.5)	97 (73.5)		21 (60.0)	14 (40.0)	
**Residence**						
Lagos	26 (21.7)	94 (78.3)	*P* = 0.48	15 (57.7)	11 (42.3)	*P* = 0.82
Osun	38 (25.3)	112 (74.7)		23 (60.5)	15 (39.5)	
**Access to SRH Information**						
No	48 (21.6)	174 (78.4)	*P* = 0.08	31 (64.6)	17 (35.4)	*P* = 0.14
Yes	16 (33.3)	32 (66.7)		7 (43.8)	9 (56.3)	
**Know modern contraceptives**						
No	12 (20.3)	47 (79.7)	*P* = 0.49	8 (66.7)	4 (33.3)	*P* = 0.57
Yes	52 (24.6)	159 (75.4)		30 (57.7)	22 (42.3)	
**Use modern contraceptives**						
Yes	54 (24.1)	170 (75.9)	*P* = 0.73	34 (63.0)	20 (37.0)	*P* = 0.17
No	10 (21.7)	36 (78.3)		4 (40.0)	6 (60.0)	

*P < 0.05

Among respondents that induced abortion of the last pregnancy, most had no education (65.0%), were aged 10–14 years (82.4%), not employed (63.6%) and lived alone (61.9%). A lower proportion of SIAs who had access to SRH information had induced abortion (43.8%); and a lower proportion of those who used a modern contraceptive reported lower proportion of pregnancy (21.7%) and induced abortion (40.0%). Significantly more respondents who were aged 10–14 years had induced abortion of the last pregnancy (*p* = 0.02).

Table 4 shows factors associated with a history of pregnancy for female SIAs. A history of ever attending school reduced the odds of unintended pregnancy when compared with those who had never attended school. This finding was observed in both the unadjusted model (OR: 0.45; 95% CI: 0.21–0.97; *p* = 0.04) and the adjusted model (AOR: 0.42; 95% CI: 0.19–0.91; p-0.03). There were non-statistically significant increased odds of unintended pregnancy among respondents aged 15–19 years, those who reported religion as less important, among those that were employed, and those living with father alone or living with both parents.

**Table 4:** Logistic regression of individual-level factors associated with unintended pregnancy among sexually active female SIAs [*n* = 270]

Variables	Model I			Model II		
Pregnancy	OR	95% C. I	*p*-value	AOR	95% C. I	*p*-value
**Age Group**						
10–14 years ^ref^	1.00	–	–	1.00	–	–
15–19 years	1.32	0.65–2.66	0.45	1.24	0.61–2.52	0.56
**Ever attended school**						
No ^**ref**^	1.00	–	–	1.00	–	–
Yes	0.45	0.21–0.97	0.04*	0.42	0.19–0.91	0.03*
**Work for pay**						
No ^**ref**^	1.00	–	–	1.00	–	–
Yes	1.50	0.78–2.88	0.23	1.42	0.73–2.75	0.30
**Importance of Religion**						
Very important ^**ref**^	1.00	–	–	1.00	–	–
Less important	1.22	0.66–2.28	0.76	1.18	0.634–2.21	0.61
**Living Arrangement**						
Living alone ^**ref**^	1.00	–	–	1.00	–	–
Living with father alone	1.11	0.31–3.92	0.88	1.12	0.80–3.27	0.18
Living with mother alone	0.68	0.21–2.24	0.53	0.71	0.22–2.35	- 0.58
Living with both parents	1.67	0.82–3.38	0.16	1.68	0.83–3.42	- 0.15
**Residence**						
Lagos ^**ref**^	1.00	–	–	1.00	–	–
Osun	1.68	0.84–3.38	0.14	1.61	0.80–3.27	0.18
**Access to SRH information**						
No ^**ref**^				1.00	–	–
Yes				1.77	0.86–3.62	0.12

*OR* Odds Ratio, *AOR* Adjusted Odds Ratio; ref.: reference category; *p* < 0.05*

Variables with *p*-value < 0.3 were included in the model to allow more relationships to be assessed considering the nature of study participants that may not follow same pattern as the general population

As shown in Table 5, being an older adolescent (15–19 years) reduced the odds of inducing abortion of the last pregnancy when compared to younger adolescents (OR: 0.12; 95% CI: 0.02–0.72; *p* < 0.02). The significance was retained in the adjusted regression model (AOR: 0.13; 95% CI: 0.02–0.77; *p* < 0.03). Also, SIAs who reported school attendance, were employed and reported religion is very important had reduced odds to induce abortion, though the associations were not statistically significant.

**Table 5:** Logistic regression of individual-level factors associated with induced abortion among sexually active female SIAs [*n* = 64]

Variables	Model I			Model II		
Induced Abortion	OR	95% CI	*p*-value	AOR	95% CI	*p*-value
**Age Group**						
10–14 years ^ref^	1.00	–	–	1.00	–	–
15–19 years	0.12	0.02–0.72	0.02*	0.13	0.02–0.77	0.03*
**Ever attended school**						
No ^**ref**^	1.00	–	–	1.00	–	–
Yes	0.77	0.11–5.56	0.80	0.69	0.09–5.50	0.73
**Work for pay**						
No ^**ref**^	1.00	–	–	1.00	–	–
Yes	0.61	0.18–2.02	0.42	0.67	0.18–2.45	0.55
**Importance of Religion**						
Very important ^**ref**^	1.00	–	–	1.00	–	–
Less important	0.61	0.18–2.02	0.42	0.62	0.18–2.21	0.43
**Living Arrangement**						
Living alone ^**ref**^	1.00	–	–	1.00	–	–
Living with father alone	0.38	0.03–4.76	0.46	0.44	0.03–5.81	0.54
Living with mother alone	0.65	0.05–7.80	0.74	0.60	0.05–7.76	0.70
Living with both parents	0.52	0.11–2.54	0.42	0.52	0.11–2.51	0.41
**Residence**						
Lagos ^ref^	1.00	–	–	1.00	–	–
Osun	2.87	0.53–15.55	0.22	3.47	0.53–22.56	0.19
**Access to SRH information**						
No ^**ref**^				1.00	–	–
Yes				0.39	0.09–1.69	0.21
**Use modern contraceptives**						
No ^**ref**^				1.00	–	–
Yes				0.57	0.08–4.07	0.58

*OR* Odds Ratio, *AOR* Adjusted Odds Ratio, *ref*. reference category; *p* < 0.05*

Variables with *p*-value < 0.3 were included in the model to allow more relationships to be assessed considering the nature of study participants that may not follow same pattern as the general population

### Qualitative results

#### Study participants characteristics

The participants’ demographic characteristics showed that most respondents were aged 15–19 years (63.6%), with a mean age of 14.8 years. Three of every five participants were Muslims (64.5%), less than half were enrolled in school (45.5%) while very few were currently employed (6.1%).

#### Themes

The themes that emerged in this study were: history of (unwanted) pregnancy, history of induced abortion, reasons for induced abortion, decision-making about procuring an abortion, place and forms of abortion, and motivations for patronizing unsafe sources.

##### History of (unwanted) pregnancy

Most FGD participants reported they knew friends that had gotten pregnant, while only a few IDI participants acknowledged they had been pregnant before. Many of the participants affirmed that unintended pregnancy was common. Also, some other participants reported a high prevalence of teenage pregnancies within their community.

*“ … none of these very young children here aborts here, cos, if they know about it, unwanted pregnancy won’t be that common in this environment … ”*

*19-year-old, FGD participant*

##### History of induced abortion

Participants acknowledged that induced abortion occurred in the population, though very few interviewees and FGD participants could share personal experiences. A majority had information about friends who induced abortion, while some reported they had a history of induced abortion. The common reason given for induced abortion was that the pregnancy was unplanned.

*“When I got pregnant ... I had a boyfriend, and I was not ready to carry the baby because I was working here in the hotel, so I went to abort it ...”*

-18-year-old, IDI participant

##### Reasons for induced abortion

In addition to the pregnancy being unwanted, other reasons highlighted by participants for inducing abortion included doubt of paternity due to multiple sexual partners, rejection of pregnancy by a sexual partner, and poor financial status.

*“What is most common is that if the young girl had sex with more than one boy, she usually goes to flush it. Even if it was just one boyfriend, they still abort it sometimes ... if she slept with only one person and the person has no job and economic power to raise a baby, he will ask her to go and flush it.*

19-year-old, FGD participant

##### Decision making about procuring an abortion

The decision-making about procuring an abortion is complex. Many participants noted that the decision to procure an abortion was mostly dependent on their relationship network, whom they can trust with their information and the social support they had.

*“what is common around here is that when some do not know what to do about the pregnancy, they either go to tell their mother, friend or the boyfriend and they can say take drugs and use it to flush it.”*

16-year-old, FGD participant

*“ … it is their boyfriends that often tell them to abort it … ”*

12-year-old, FGD participant

*“ … for me, it is D&C that I did, my aunty is a nurse, she told me that it is the only way, that I should not take drugs … she took me to the hospital, she said I can have side effect from using drugs … ”*

17-year-old, FGD participant

*“... it was my friend that took me to a guy's place, and he gave me some drugs it was a little painful, but at least I was not pregnant anymore.”*

*-*18-year-old, FGD, participant

##### Place and methods of inducing abortion

The places visited to procure an abortion were private hospitals, chemist shops and traditional herbal practitioners. Methods for inducing abortion include the use of medications, local herbal and drug mixtures, use of coarse substances and insertion of metal objects. Other “high-risk.” methods of inducing abortion were the use of objects such as cloth hangers and drinking of poly-substances made up of two or more substances. Remarkably very few participants were aware of how to procure a medical abortion, and neither did anyone identify public hospitals as a place to procure an abortion.

*“we have heard about alabukun [analgesic] and ampiclox [antibiotic]; we know they are being used to terminate pregnancy”*

13-year-old, FGD participant

*“I have not had an abortion since I have not been pregnant before, but, one that happened in my presence. Actually, the person's womb was ruined in the end. For someone to use potash and blue, the blue dye used for clothes ... there was blood all over the bathroom that day”*

19-year-old, IDI participant

##### Motivations for patronizing unsafe sources

Multiple factors were highlighted as the reason for procuring unsafe abortion. These include the high cost of procuring abortion in private health facilities, the illegality of abortion in the country, cultural barriers, the unfriendly environment towards young people seeking SRH services, fear of meeting someone known in a formal health setting and lack of privacy. The gestational age of the pregnancy may however, be a driver to seek expert care as indicated in one of the excerpts below:

*“why the private hospital is the most commonplace is that if they go to government hospitals, they might meet someone who will advise them to keep it. If they go to private clinics, they will say that they only need to pay and the services will be carried out”*

19-year-old, FGD participant

*“...there are people that go to a normal hospital for abortion, but the doctor may ask them not to disclose his/her identity. The doctor will do the abortion for them, but it’s not cheap. It’s really expensive. If the pregnancy is like five months, you know by then the baby is well formed. At that stage, you need someone competent. So, abortions can be done in different ways.”*

16-year-old, FGD participant

## Discussion

The study is the first to provide large-scale data on SIAs in Nigeria and one of the few studies on street-involved young people in Africa [21–23; 9] with a focus on female adolescents. The findings show that one in every five sexually active SIAs had been pregnant while half of the pregnancies ended in induced abortion. The factors associated with unintended pregnancy differ slightly from those associated with induced abortion. Having attended school reduced the risk of having an unintended pregnancy while being an older adolescent reduced the risk of inducing an abortion. The main reason for inducing abortion was having an unwanted pregnancy. Abortions were mainly induced using unsafe drugs and procedures. Pregnant adolescents resort to the use of unsafe procedures to procure abortion because of the lack of safe spaces and the absence of a supportive environment for pregnant adolescents.

The strengths of the study include a large number of participants, the recruitment of study participants from two states in Southwest Nigeria with different cosmopolitan features, and the recruitment of young adolescents (10–14 years old). The use of a mixed method enabled us to explore the perspectives of SIAs further. The study however, had a few limitations. Though the study sample was recruited using methods appropriate for the study population and efforts were made to ensure recruitment from different clusters of the study population, it may not be representative of Southwestern Nigeria, and it is not representative of Nigeria. Also, the study did not have a comparative arm, hence differences in the pregnancy rates between SIAs and other adolescents could not be analysed. The study included only four IDI from female adolescents 10–19-years-old. The inclusion of the transcripts of three FGD conducted with 33 adolescents 10–19-years-old addressed the concerns that the limited number of IDI conducted for the target population may have raised. Including the four IDI in the study analysis enriched the study findings.

Despite these limitations, this study had further strengthened existing evidence that education improves health outcomes, including a reduced risk of unwanted pregnancy. In many low- and middle-income countries, a low level of education is a significant predictor of teenage and unwanted pregnancies [36–39]. Prior studies in Nigeria had reported similar findings [37, 40, 41]. Higher education attainment improves access to information that can be protective. It also improves individuals’ financial status and the ability to procure preventive services [42].

Though the study finding may indicate that access to SRH information may not be protective of unwanted pregnancy in this study population, this finding needs to be interpreted with caution. We opine that the findings may indicate that those who accessed SRH may likely be those that needed to manage their pregnancies rather than those who access services for preventive care. Further studies are needed to explain the findings.

Older adolescents are less likely to induce abortion because many may be considered old enough to be in marital unions, so pregnancies are welcome. The 2018 Nigeria Demographic Health Survey report [43] showed that 19% of older adolescents aged 15–19 years have begun childbearing and much earlier so with rural than urban teenagers (27% versus 8%). An earlier study among street-connected kids in Kenya [44] shows pregnancy is viewed as a sense of identity, hope and source of income. Hence, the need to induce abortion may be less so for older adolescents but a pressing need for younger adolescents concerned about stigma [45–47]. This stigma may also be the reason for procuring abortion among those that are religious, as found in this study. Prior studies in Nigeria had indicated that religiosity is protective of unwanted pregnancy [48, 49]. This study indicates that when teenagers get pregnant, being religious may increase the likelihood of induced abortion, perhaps to save face as pregnancy out of wedlock is against religious norms and causes shame, stigma and distress [50].

The high rate of procured abortion may indicate a high rate of unmet contraception needs for adolescents in Nigeria [21, 51]. However, providing access to contraceptives may not necessarily translate to increased use; and when contraception use is initiated, the discontinuation rate is high among adolescents [52]. The unmet contraception needs may be higher for SIAs, though our study was unable to highlight this in the absence of a comparator. Future studies are needed to help identify how large the unmet contraception need of SIAs is compared with other adolescents, as this will help highlight the peculiar contraception needs of SIAs.

The interplay of various decision-making determinants regarding pregnancy termination among SIAs reflects the complexity of this issue. As indicated in the qualitative excerpts, factors such as the timing of the pregnancy, financial constraints, paternity concerns, status of the SIA’s relationship with her partner, parental support, and societal expectations are important influences for deciding to procure an abortion. When the decision to procure an abortion is made, termination by non-medical persons becomes a choice for reasons similar to those reported by Wachira et al. [44] for street-connected children and youth in Kenya. The criminalization of abortion in Nigeria [53] and the limited availability of and accessibility to SRH services for young people in the country [54], especially the disadvantaged and vulnerable population sub-group, further contribute to the high prevalence of unsafe abortions.

## Conclusion

The study highlights that the factors associated with an unwanted pregnancy and those associated with induced abortion differ among female SIAs, 10–19-years-old in South-west Nigeria. The prevalence of unwanted pregnancy and induced abortion is high. These findings highlight the need for further studies to identify the peculiar needs of SIAs to help inform policy formulations and program development that will address these needs in Nigeria.

## Data Availability

The datasets used and/or analysed during the current study available from the corresponding author on reasonable request.

## References

[1] Ganatra B, Tuncalp O, Johnston HB, Johnson BR Jr, Gulmezoglu AM, Temmerman M. From concept to measurement: operationalizing WHO's definition of unsafe abortion. SciELO. Public Health. 2014. 10.2471/BLT.14.136333. 10.2471/BLT.14.136333 PMC3949603 24700971

[2] Shah IH, Åhman E (2012). Unsafe abortion differentials in 2008 by age and developing country region: high burden among young women. Reprod Health Matters.

[3] Preventing unsafe abortion. Evidence brief. World Health Organization. 2019. WHO/RHR/19.21. Available from: https://apps.who.int/iris/bitstream/handle/10665/329887/WHO-RHR-19.21-eng.pdf

[4] Ganatra B, Gerdts C, Rossier C, Johnson BR, Tunçalp Ö, Assifi A (2017). Global, regional, and subregional classification of abortions by safety, 2010–14: estimates from a Bayesian hierarchical model. Lancet.

[5] Assifi AR, Kang M, Sullivan EA, Dawson AJ (2020). Abortion care pathways and service provision for adolescents in high-income countries: a qualitative synthesis of the evidence. PLoS One.

[6] Guttmacher Institute (2018). Induced abortion worldwide: Global Incidence and Trends.

[7] Darroch JE, Woog V, Bankole A, Ashford LS (2016). Adding it UP: costs and benefits of meeting the contraceptive needs of adolescents.

[8] World Health Organisation. Unsafe abortion incidence and mortality - Global and regional levels in 2008 and trends 2012. Available from: https://apps.who.int/iris/bitstream/handle/10665/75173/WHO_RHR_12.01_eng.pdf?sequence=1&isAllowed=y

[9] Haddad LB, Nour NM (2009). Unsafe abortion: unnecessary maternal mortality. Rev Obstet Gynecol.

[10] United Nations General Assembly (2015). Sustainable development goals.

[11] UN WOMEN. SDG 3: Ensure healthy lives and promote well-being for all at all ages. Accessed 2 Dec 2022. Available at: https://www.unwomen.org/en/news/in-focus/women-and-the-sdgs/sdg-3-good-health-well-being

[12] IPM. Women’s Sexual and Reproductive Health & the SDGs 8405 Colesville Rd., Ste. 600, Silver Spring, MD 20910 USA2018 [November 11,2019]. Available from: https://www.ipmglobal.org/why-microbicides/womens-sexual-reproductive-health-sdgs.

[13] Atuyambe LM, Kibira SP, Bukenya J, Muhumuza C, Apolot RR, Mulogo E (2015). Understanding sexual and reproductive health needs of adolescents: evidence from a formative evaluation in Wakiso district, Uganda. Reprod Health.

[14] Coker TR, Sareen HG, Chung PJ, Kennedy DP, Weidmer BA, Schuster MA (2010). Improving access to and utilization of adolescent preventive health care: the perspectives of adolescents and parents. J Adolesc Health.

[15] Johnson KM, Dodge LE, Hacker MR, Ricciotti HA (2015). Perspectives on family planning services among adolescents at a Boston community health center. J Pediatr Adolesc Gynecol.

[16] Decker MJ, Atyam TV, Zárate CG, Bayer AM, Bautista C, Saphir M (2021). Adolescents’ perceived barriers to accessing sexual and reproductive health services in California: a cross-sectional survey. BMC Health Serv Res.

[17] UNFPA (2013). UNFPA strategy on adolescents and youth: towards realizing the full potential of adolescents and youth accessed.

[18] UNICEF (1985). Worksheet for the regional operating plan for abandoned and street children.

[19] Panter-Brick C. Street children, human rights, and public health: a critique and future directions. Annu Rev Anthropol. 2002:147–71.

[20] (UNFPA) UNPF (2007). UNFPA Framework for Action on Adolescents & Youth.

[21] Owoaje E, Uchendu O (2009). Sexual risk behaviour of street youths in South West Nigeria. East Afr J Public Health.

[22] Brhane T, Assefav B, Birhan N (2014). Reproductive health behaviour of street youth and associated factors in Gondar city, Northwest Ethiopia. Int J Med Biomed Res.

[23] Edewor PA. Homeless children and youths in Lagos, Nigeria: their characteristics, street life and sexual behaviour. Mediterranean journal of Social Sciences. 2014; 5(1):537–545.

[24] Olugbenga-Bello AI, Ilori OR, Idowu T (2022). Street youths: reproductive health risk status, reproductive health challenges and barriers to health services utilization in a southwestern City. Nigeria Afri Health Sci.

[25] Olaleye AO, Obiyan MO, Folayan MO (2020). Factors associated with sexual and reproductive health behaviour of street-involved young people: findings from a baseline survey in Southwest Nigeria. Reprod Health.

[26] Aransiola JO, Zarowsky C (2014). Street children, human trafficking and human security in Nigeria: competing discourses of vulnerability and danger. Afr Popul Stud.

[27] Isiugo-Abanihe UC, Olajide R, Nwokocha E, Fayehun F, Okunola R, Akingbade R (2015). Adolescent sexuality and life skills education in Nigeria: to what extent have out-of-school adolescents been reached?. Afr J Reprod Health.

[28] World Health Organisation (2018). Adolescents: health risks and solutions.

[29] Population Reference Bureau (2019). Youth Family Planning Policy Scorecard: Measuring Commitment to Effective Policy and Program Intervention.

[30] Federal Ministry of Health (2014). Guidelines for young persons’ participation in research and access to sexual and reproductive health services.

[31] Mugove K, Lincoln H (2015). Why children leave their homes for the streets? The case of Harare. Int J Sci Res Publ.

[32] Blair E (2015). A reflexive exploration of two qualitative data coding techniques. J Methods Measure Soc Sci.

[33] Strauss A, Corbin J (1998). Basics of qualitative research techniques.

[34] Fereday J, Muir-Cochrane E (2006). Demonstrating rigor using thematic analysis: a hybrid approach of inductive and deductive coding and theme development. Int J Qual Methods.

[35] Saldaña J. The coding manual for qualitative researchers: Sage; 2015.

[36] Kassa GM, Arowojolu A, Odukogbe A, Yalew AW (2018). Prevalence and determinants of adolescent pregnancy in Africa: a systematic review and Meta-analysis. Reprod Health.

[37] Kunnuji MO, Eshiet I, Nnorom CC (2018). A survival analysis of the timing of onset of childbearing among young females in Nigeria: are predictors the same across regions?. Reprod Health.

[38] Odimegwu C, Mkwananzi S (2016). Factors associated with teen pregnancy in sub-Saharan Africa: a multi-country cross-sectional study. Afr J Reprod Health.

[39] Faghihzadeh S, Babaee Rochee G, Lmyian M, Mansourian F, Rezasoltani P (2003). Factors associated with unwanted pregnancy. J Sex Marital Ther.

[40] Izugbara C (2015). Socio-demographic risk factors for unintended pregnancy among unmarried adolescent Nigerian girls. S Afr Fam Pract.

[41] Isa AI, Gani IOO (2012). Socio-demographic determinants of teenage pregnancy in the Niger Delta of Nigeria. Open J Obstet Gynecol.

[42] Zimmerman E, Woolf SH (2014). Understanding the relationship between education and health: discussion paper.

[43] National Population Commission (NPC) I (2019). Nigeria Demographic and Health Survey 2018.

[44] Wachira J, Kamanda A, Embleton L, Naanyu V, Ayuku D, Braitstein P (2016). ‘Pregnancy has its advantages’: the voices of street connected children and youth in Eldoret, Kenya. PLoS One.

[45] Levandowski BA, Kalilani-Phiri L, Kachale F, Awah P, Kangaude G, Mhango C (2012). Investigating social consequences of unwanted pregnancy and unsafe abortion in Malawi: the role of stigma. Int J Gynecol Obstet.

[46] Dahlbäck E, Maimbolwa M, Kasonka L, Bergström S, Ransjö-Arvidson A-B (2007). Unsafe induced abortions among adolescent girls in Lusaka. Health Care Women Int.

[47] Munakampe MN, Zulu JM, Michelo C (2018). Contraception and abortion knowledge, attitudes and practices among adolescents from low and middle-income countries: a systematic review. BMC Health Serv Res.

[48] Fatusi AO, Blum RW (2008). Predictors of early sexual initiation among a nationally representative sample of Nigerian adolescents. BMC Public Health.

[49] Akanbi ST, Adika LO (2015). Self-concept, maternal attachment and religiosity as predictors of sexual debut among school-going adolescents in Oyo state, Nigeria. Afr J Psychol Stud Soc Issues.

[50] Adamczyk A. The effects of religious contextual norms, structural constraints, and personal religiosity on abortion decisions. Soc Sci Res (2008). 37:16. 10.1016/j.ssresearch.2007.09.003. 10.1016/j.ssresearch.2007.09.003 19069064

[51] Otoide VO, Oronsaye F, Okonofua FE. Why Nigerian adolescents seek abortion rather than contraception: evidence from focus-group discussions. Int Fam Plan Perspect. 2001:77–81. 10.2307/2673818.

[52] Chandra-Mouli V, Parameshwar PS, Parry M, Lane C, Hainsworth G, Wong S (2017). A never-before opportunity to strengthen investment and action on adolescent contraception, and what we must do to make full use of it. Reprod Health.

[53] Okagbue I (1990). Pregnancy termination and the law in Nigeria. Stud Fam Plan.

[54] Onukwugha FIHM, Magadi MA (2019). Views of service providers and adolescents on use of sexual and reproductive health services by adolescents: a systematic review. Afr J Reprod Health.

